# Digging deeper: quality of patient-provider communication across Hispanic subgroups

**DOI:** 10.1186/1472-6963-9-240

**Published:** 2009-12-21

**Authors:** Lorraine S Wallace, Jennifer E DeVoe, Edwin S Rogers, Joanne Protheroe, Gillian Rowlands, George E Fryer

**Affiliations:** 1 University of Tennessee Graduate School of Medicine, Department of Family Medicine, Knoxville, Tennessee, USA; 2 Oregon Health & Science University, Department of Family Medicine, Portland, Oregon, USA; 3 University of Manchester, Manchester, UK; 4 London South Bank University, Institute of Primary Care and Public Health, London, UK; 5 University of Arkansas for Medical Sciences, Department of Pediatrics, Little Rock, Arkansas, USA

## Abstract

**Background:**

Recent research suggests that ethnic subgroup designation plays an important role in health-related disparities among Hispanics. Our objective was to examine the influence of Hispanics' self-reported ethnic subgroup designation on perceptions of their health care providers' communication behaviors.

**Methods:**

Cross-sectional analysis of the 2005 Medical Expenditure Panel Survey (MEPS). Participants included non-institutionalized Hispanics (n = 5197; US population estimate = 27,070,906), aged ≥18 years, reporting visiting a health care provider within the past 12 months. Six (n = 6) items were used to capture respondents' perceptions of their health care providers' communication behaviors.

**Results:**

After controlling for socio-demographic covariates, compared to Other Hispanics (reference group), very few differences in perceptions of health care providers communication emerged across ethnic subgroups. Puerto Ricans were more likely to report that their health care provider "always" showed respect for what they had to say (OR = 2.16, 95% CI 1.16-4.03). Both Puerto Ricans (OR = 2.28, 95% CI 1.06-4.92) and Mexicans (OR = 1.88, 95% CI 1.02-3.46) were more likely to indicate that their health care provider "always" spent enough time with them as compared to Other Hispanics.

**Conclusions:**

We observed very few differences among Hispanics respondents in their perceived quality of interactions with health care providers as a function of their ethnic subgroup designation. While our findings somewhat contradict previous research, they do suggest that other underlying factors may influence the quality of perceived interactions with health care providers.

## Background

Hispanic Americans now represent 15% of the total US population [1]. Over the past decade, studies have consistently documented a wide array of health-related disparities between Hispanics and non-Hispanic whites [2]. For instance, using data from the 2001 National Health Interview Survey, McGruder and colleagues [3] found that Hispanics were less aware of heart attack symptoms and the need to call for emergency help if someone near them had symptoms than were whites. In a population-based study of respondents residing in ten different states, Hispanics reported receipt of fewer routine preventive services (e.g., colorectal cancer screening and cholesterol testing) and less access to physicians (e.g., had difficulty paying for a clinical examination) as compared to their non-Hispanic white counterparts [4]. Furthermore, evidence suggests that ethnic minorities, including Hispanics, are not only more likely to report feelings of health care provider discrimination, but also poorer health status as compared to non-Hispanic whites [5].

As health-related disparities are further examined, several researchers have raised concerns that not all Hispanics experience the same health problems. Mainous et al. [6] found significant differences in the quality of diabetes care across Hispanic ethnic subgroups. For example, Mexicans were less likely than Puerto Ricans to know about glycosylated hemoglobin. Similarly, among a nationally representative sample of Hispanic women in the United States, those of Mexican or South/Central American origin were more likely than Puerto Ricans or Cubans to lack health insurance coverage [7]. Interestingly, Jerant [8] found that Mexicans not only reported better self-reported health status as compared to other Hispanics, but whites as well. Collectively, findings from these studies indicate that marked discrepancies exist between Hispanic ethnic subgroups. Therefore, treating Hispanics as one homogenous grouping may mask important differences.

Because Hispanics are a heterogeneous group, it is important to identify underlying factors that may indirectly be contributing to compromised access and worse disease. The quality of communication dynamics during the clinical encounter between the patient and health care provider could be one such factor contributing to observed health-related differences in Hispanic subgroups. For instance, perceived quality of patient-health care provider communication has shown to differ significantly between Hispanics and non-Hispanic whites. In a recent study of adults from three states, Hispanics were more likely to report that health care providers did not carefully listen to them as compared to non-Hispanics; however, respondent ethnicity was not related to differences in other communication variables [9]. Further, among a nationally representative sample of civilian adults, Wallace et al. [10] found somewhat paradoxical results related to patient perceptions of health care provider communication skills. Hispanics were more likely to report that some aspects of patient-health provider communication were of high quality (e.g., provider "always" listened to them) while others were not (e.g., provider did not "always" give them control over treatment options) as compared to non-Hispanic whites.

The quality of patient-healthcare provider communication within the Hispanic population exclusively has just begun to be explored. Recently, the influence of language preference--English versus Spanish--on Hispanics' perceptions of their healthcare providers' communication behaviors was examined [11]. Overwhelming, English responders were more likely to report more positive interactions with health care providers (e.g., provider "always" explained things so that they understood and "always" asked them to help make decisions) than Spanish responders.

However, to our knowledge, no studies have examined perceptions of health care communication among members of Hispanic subgroups. To address this gap in the literature, this study examined the influence of Hispanics' self-reported ethnic subgroup designation--Central/South American, Dominican, Cuban, Puerto Rican, Mexican, Other--on perceptions of their health care providers' communication behaviors during clinical encounters.

## Methods

### Design and Subjects

We conducted an analysis of the 2005 Medical Expenditures Panel Survey Household Component file (MEPS-HC) [12]. The MEPS-HC, a nationally representative sample of civilian non-institutionalized US adults, utilizes a stratified multi-stage area probability design in which certain groups (e.g., limited income, racial minorities) are over-sampled. With the aid of computer-assisted personal interviewing technology, MEPS-HC respondents are interviewed in their homes five times over a two-year period. The interviews are conducted in the respondent's language of choice. Respondents are queried on such topics as socio-demographic characteristics, self-reported health status, health insurance coverage, and use of, access to, and satisfaction with healthcare providers and services. The response rate for the full-year 2005 MEPS-HS was 61.3% [13]. Subjects included in this study were Hispanic adults, ≥18 years of age, who had visited a healthcare provider within the 12 months preceding data collection.

### Variables

The predictor variable was respondents' self-reported ethnic subgroup designation (Central/South American, Dominican, Cuban, Puerto Rican, Mexican, or Other). Health services utilization models [14-16] were used to guide the selection of relevant socio-demographic variables for our multivariate models, including: sex, age (at time of interview), selected language of interview (English versus Spanish), place of residence (metropolitan statistical area [MSA]) categorization, family income (adjusted for family size), completion of high school (head of household), census region, health insurance status, and usual source of care (USC).

Six (n = 6) MEPS items were identified and subsequently used as outcome variables to determine respondents' perceptions of healthcare providers' communication behaviors during clinical encounters. Responses to these six items were reported on a 4-point Likert-type scale (always, usually, sometimes, never). All respondents reporting a doctor's office or clinic visit within the 12 months preceding data collection (n = 5197) answered the following items: (1) "*How often did providers listen carefully to you?*"; (2) "*How often did providers explain things so you understood?*"; (3) "*How often did providers show respect for what you had to say?*"; and (4) "*How often did providers spend enough time with you?*" Additionally, those reporting having an identified USC (n = 2832) responded to the following two items: (5) "*If there were a choice between treatments, how often would your provider at your USC ask you to help make the decision?*" and (6) "*How often does your provider at your USC show respect for treatments?" *These exact [10,11] and similar [9,17] items have been used previously to gauge patients' perceptions of the quality of interactions they had with health care providers.

### Statistical Analyses

Because the MEPS is based on a complex sampling design that makes it representative of the civilian, non-institutionalized US population, SUDAAN (Research Triangle Institute, Research Triangle Park, NC) statistical software (Release 9.0.3) was used to account for the weighting and complex sampling design. Alpha was set at .05 *a priori *for all tests of statistical significance.

Multiple logistic regression analyses were performed to assess the association of respondents' self-reported ethnic subgroup designation on perceptions of their recent healthcare interactions, while simultaneously controlling for the effect of all covariates (i.e., socio-demographic variables). For the purposes of multivariate logistic regression analyses, responses to these six MEPS items were dichotomized as "always" and "not always" (usually/sometimes/never). Results of the multiple logistic regression models are reported in adjusted odds ratios (ORs) with corresponding 95% confidence intervals (CIs). For the purposes of our analyses, Other Hispanics served as the reference group. This strategy--using one Hispanic subgroup as the reference group--has been used by other researchers conducting similar studies [6-8].

## Results

In 2005, 5197 Hispanic MEPS-HC respondents (weighted sample size = 27,070,906), ≥18 years of age, reported visiting a healthcare provider within the 12 months preceding data collection. Socio-demographic and related characteristics of Hispanic respondents--by ethnic subgroup designation--are presented in Table 1. Males and females were represented fairly equally except among Dominicans, where there were significantly fewer males. Overall, Cubans tended to be older and more likely to have a high school education and a higher family income. A large percentage of respondents from all subgroups lived in an urban area. Puerto Ricans were most likely to have private health insurance (57.3%) versus Dominicans who were least likely (32.5%). Lack of health insurance was highest among Mexicans and Central/South Americans. Cubans were most likely to have a USC (68.5%), compared with Central/South Americans who were the least likely (49.7%).

**Table 1:** Sociodemographic and related characteristics of Hispanic respondents by ethnic subgroup

	Ethnic Subgroup					
	
Sociodemographic and Related Characteristics	Mexican **[n = 3794]**^**a**^ **(%)**^**b**^	Puerto Rican **[n = 336]**^**a**^ **(%)**^**b**^	Cuban **[n = 172]**^**a**^ **(%)**^**b**^	Dominican **[n = 119]**^**a**^ **(%)**^**b**^	Central/ South American **[n = 634]**^**a**^ **(%)**^**b**^	Other **[n = 142]**^**a**^ **(%)**^**b**^
Sex						
Male	52.9	43.5	53.0	30.8	52.6	50.6
Female	47.1	56.5	47.0	69.2	47.4	49.4

Age Group						
18-24 Years	18.5	19.1	6.5	13.8	16.9	17.4
25-44 Years	50.7	42.9	38.1	50.8	52.9	47.3
45-64 Years	22.7	30.7	31.0	27.9	25.0	25.0
≥65 Years	8.1	7.3	24.4	7.5	5.2	10.3

Interview Conducted in English						
Yes	48.9	65.7	39.2	42.5	31.0	58.9
No	51.1	34.3	60.8	57.5	69.0	41.1

Place of Residence						
MSA	92.1	95.1	99.4	99.9	97.0	94.9
Non-MSA	7.9	4.9	0.6	0.1	3.0	5.1

Family Income						
Poor	18.3	18.6	9.1	31.4	14.5	12.0
Near Poor	8.4	6.9	7.7	9.4	5.3	4.0
Low Income	22.2	14.1	23.7	15.1	22.6	16.6
Middle Income	32.8	30.2	27.1	33.2	33.9	37.6
High Income	18.3	30.2	32.4	10.9	23.7	29.8

High School Graduate						
Yes	50.1	65.9	75.4	60.7	57.1	76.4
No	49.9	34.1	24.6	39.3	42.9	23.6

Census Region						
Northeast	1.6	58.3	15.6	68.9	31.1	28.5
Midwest	9.2	7.7	4.1	3.6	2.9	5.4
South	35.2	24.8	78.1	27.5	39.0	44.0
West	54.0	9.2	2.2	0.0	27.0	22.1

Health Insurance						
Any Private	45.7	57.3	54.9	32.5	50.8	45.3
Public	16.3	26.3	27.6	37.6	11.6	18.6
Uninsured	38.0	16.4	17.5	29.9	37.6	36.1

*Usual Source of Care*						
*Yes*	56.9	78.7	68.5	64.6	49.7	66.5
*No*	43.1	21.3	31.5	35.4	50.3	33.5

MSA = metropolitan statistical area

^a^Unweighted counts represent the total number of MEPS-HC respondents with a positive person weight who visited a healthcare provider in the past year.

^b^Weighted percentages. To derive nationally-representative population estimates, each respondent record from the MEPS was weighted according to person-level weights provided by the data-collection agency.

**Figure 1:**
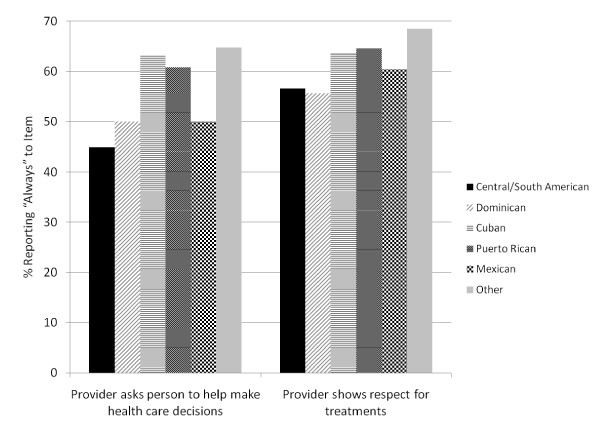
**Percentage of respondents with a self-reported usual source of care, by ethnic subgroup, reporting that their healthcare provider "always" asked them to help make health care decisions and showed respect for treatments**.

**Figure 2:**
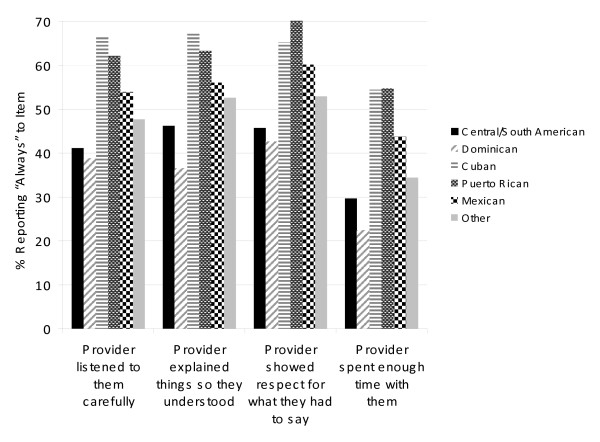
**Percentage of respondents, by ethnic subgroup, reporting that their health care provider "always" listened to them carefully, explained things so they understood, showed respect for what they had to say, and spent enough time with them**.

As displayed in Figure 1 and Table 2, between a third and two-thirds of Hispanic respondents reported that their healthcare provider "always" listened to them carefully, explained things so that they understood, and showed respect for what they had to say. Collectively, respondents were less inclined to indicate that their healthcare provider "always" spent enough time with them. Among those with a USC, approximately 50-60% of respondents overall reported that their healthcare provider "always" asked them to help make healthcare decisions and showed respect for treatments (see Figure 2 and Table 2).

**Table 2:** Hispanic Respondents perceptions of healthcare providers' communication behaviors by ethnic subgroup

MEPS Items Pertaining to Healthcare Providers Communication Behaviors	Adjusted OR **(95% CI)**^**a**^
***Provider listened to them carefully***^**b**^	
Central/South American	0.92 (0.46, 1.84)
Dominican	0.95 (0.35, 2.58)
Cuban	2.03 (0.91, 4.53)
Puerto Rican	1.94 (0.93, 4.05)
Mexican	1.36 (0.71, 2.60)
Other	1.00

***Provider explained things so they understood***^**b**^	
Central/South American	0.90 (0.50, 1.61)
Dominican	0.72 (0.29, 1.79)
Cuban	1.56 (0.72, 3.36)
Puerto Rican	1.67 (0.86, 3.27)
Mexican	1.18 (0.65, 2.13)
Other	1.00

***Provider showed respect for what they had to say***^**b**^	
Central/South American	0.88 (0.53, 1.45)
Dominican	0.86 (0.30, 2.47)
Cuban	1.31 (0.59, 2.87)
Puerto Rican	**2.16 (1.16, 4.03)**
Mexican	1.52 (0.91, 2.53)
Other	**1.00**

***Provider spent enough time with them***^**b**^	
Central/South American	1.06 (0.54, 2.08)
Dominican	0.65 (0.31, 1.35)
Cuban	2.03 (0.88, 4.66)
Puerto Rican	**2.28 (1.06, 4.92)**
Mexican	**1.88 (1.02, 3.46)**
Other	**1.00**

***Provider asks person to help make health care decisions***^**c**^	
Central/South American	**0.46 (0.23, 0.95)**
Dominican	0.43 (0.13, 1.49)
Cuban	0.95 (0.37, 2.39)
Puerto Rican	0.74 (0.37, 1.49)
Mexican	0.63 (0.33, 1.20)
Other	**1.00**

***Provider shows respect for treatments***^**c**^	
*Central/South American*	0.70 (0.34, 1.41)
*Dominican*	0.47 (0.20, 1.12)
*Cuban*	0.76 (0.30, 1.92)
*Puerto Rican*	0.69 (0.31, 1.54)
*Mexican*	0.84 (0.43, 1.66)
*Other*	1.00

MEPS = Medical Expenditures Panel Survey, OR = odds ratio, CI = confidence interval

^a^Statistical significance indicated by bold highlights.

^b^Estimates pertain to Hispanic civilian, non-institutionalized U.S. adults who visited a healthcare provider in the past year. Multiple logistic regression models adjusted for sex, age, selected language of interview, place of residence, family income, educational attainment, census region, health insurance status, and usual source of care.

^c^Estimates pertain to Hispanic civilian, non-institutionalized U.S. adults who reported having a usual source of care in 2005. Multiple logistic regression models adjusted for sex, age, selected language of interview, place of residence, family income, educational attainment, census region, and health insurance status.

After controlling for the effects of all socio-demographic and related characteristics outlined in Table 1, as compared to Other Hispanics, very few differences emerged in perceptions of healthcare providers' communication behaviors across ethnic subgroups. Of note, as compared to Other Hispanics (reference group = 1.00), Puerto Ricans were more likely to report that their healthcare provider "always" showed respect for what they had to say (OR = 2.16, 95% CI 1.16-4.03). Both Puerto Ricans (OR = 2.28, 95% CI 1.06-4.92) and Mexicans (OR = 1.88, 95% CI 1.02-3.46) were more likely to indicate that their healthcare provider "always" spent enough time with them as compared to Other Hispanics. As compared to Other Hispanics, Central/South Americans were less likely to report that their healthcare provider "always" asked them to help make healthcare decisions (OR = 0.46, 95% 0.23-0.95).

## Discussion

This is the first study, to our knowledge, where influence of Hispanics' self-reported ethnic subgroup designation--Central/South American, Dominican, Cuban, Puerto Rican, Mexican, Other--on perceptions of their health care providers' communication behaviors during clinical encounters was examined. While significant health-related differences across Hispanic subgroups have been reported previously [6-8], the experiences among members of various Hispanic subgroups related to self-reported interactions with health care providers were more alike than they were different. While our findings somewhat contradict previous research, they do suggest that other underlying factors besides ethnic subgroup may influence the quality of perceived interactions with health care providers.

Importantly, established health services utilization models [14-16] were used to guide our selection of nine socio-demographic variables for inclusion in our multivariate models, including language preference for interview. Hence, our findings indicate that the few differences between ethnic subgroups we noted could be explained by other socio-demographic variables not included in our multivariate models. Degree of acculturation could perhaps be an important construct that may differentiate Hispanics' perceived interactions with health care providers. While acculturation can be defined and measured different ways, language preference (English or Spanish) has been used as a proxy for acculturation in previous studies [6,18]. Therefore, when possible it may advantageous to measure acculturation using a more robust instrument such the *Short Acculturation Scale for Hispanics *which includes measures of not only language use, but also items pertaining to media use and ethnic social relations [19].

Reported measures of "respect" (for Puerto Ricans) and adequate time spent in the medical encounter were highest (for Mexicans and Puerto Ricans) among groups which tended to have the most confident use of English in the survey. Also, it must be noted that the Puerto Rican subgroup were most likely to have private health insurance. Thus, there may be other factors related to acculturation that differs among subgroups, but could not be completely captured in our language covariate. Further, although we controlled for whether or not respondents had a USC, the significant finding among Central/South Americans who felt less involved in health care decision-making may be related to an unmeasured factor that also made them least likely to report receiving care from a consistent, usual source.

While we noted very few significant differences among Hispanic ethnic subgroups, a large proportion of respondents (≈30-70%) overall reported that health care providers did not "always" listen to them carefully, explain things so that they understood, show respect for what they had to say, spend enough time with them, ask them to help make health care decisions, and/or show respect for treatments. Unfortunately, these findings are troubling given the importance of and the movement toward patient-centered care [20,21]. Notably, our findings are quite similar to analyses where these exact [10,11] and similar [9,17] type items were used to assess respondents' perceptions of their health care providers' communication behaviors in large population-based studies. However, when asked to assess their health care providers' communication skills shortly after a consultation, Makoul et al. [22] found that between 63% and 84% of patients gave the most positive answer possible. These observed differences could be attributed to differences in study populations and/or the timing of questions.

Based on our findings, the Hispanic population appears be a more cohesive group than has been assumed, at least in terms of their reported perceptions about health care communication. In contrast to previous studies that have found major differences among Hispanic subgroups related to accessing health care services [6-8], this study found few differences, which may suggest that some of the reported differences in access are not necessarily related to how well these groups perceive communication between themselves and their health care providers. It also suggests a need for future studies to examine each ethnic subgroup in more depth. For example, qualitative methodologies--focus groups, in-depth interviews--could provide valuable insight into underlying factors associated with access barriers and how these may or may not relate to perceived perceptions of health care providers' communication behaviors.

This study has several limitations. First, although the MEPS is representative of the civilian, non-institutionalized US population, the cross-sectional format limits causality. Second, as with all observational studies that rely on self-reports, response bias remains a possibility. Third, because of the nature of secondary analyses we could not revise MEPS-HC items that pertained to the objectives of our particular study.

## Conclusion

In conclusion, we found few differences in perceptions of patient-provider communication among different Hispanic subgroups. While further study is needed, our findings suggest that responses from one large combined Hispanic ethnic group will usually represent the subgroups reliably--as long as several confounding variables are considered simultaneously (e.g., language preference)--when considering their perceptions about health care communication.

Ensuring equity of health, and access to high quality health information and health care, are issues common to most industrialized nations. Our study highlights the need to undertake both quantitative and qualitative exploration of health care provider communication amongst Hispanic subgroups; ensuring the issues relating to health care advice and health care access to be identified and addressed for as many people as possible.

## Competing interests

The authors declare that they have no competing interests.

## Authors' contributions

LW conceived of the study and drafted the manuscript. JD participated in the design of the study and helped in drafting the manuscript. ER participated in the design of the study and helped in drafting the manuscript. JP gave the research careful advice and revised the manuscript. GR gave the research careful advice and revised the manuscript. GF participated in the design of the study and conducted all statistical analyses. All authors read and approved the final manuscript.

## Pre-publication history

The pre-publication history for this paper can be accessed here:

http://www.biomedcentral.com/1472-6963/9/240/prepub
